# Genetic Structure of *Monochamus alternatus* (Hope) in Qinling‐Daba Mountains and Expansion Trend: Implications for Pest Prevention and Management

**DOI:** 10.1002/ece3.70373

**Published:** 2024-10-08

**Authors:** Jingyu Qi, Junke Nan, Xiaogu Zhao, Chaoqiong Liang, Jiangbin Fan, Hong He

**Affiliations:** ^1^ Key Laboratory of National Forestry and Grassland Administration for Control of Forest Biological Disasters in Western China, College of Forestry Northwest A&F University Yangling China; ^2^ Key Laboratory of Plant Protection Resources and Pest Management of Ministry of Education, College of Plant Protection Northwest A&F University Yangling Shaanxi China; ^3^ Shaanxi Academy of Forestry Xi'an China

**Keywords:** future predictions, mitochondrial genes, pine wilt disease, population differentiation, vector insect

## Abstract

Pine wilt disease (PWD), caused by *Bursaphelenchus xylophilus*, severely threatens global pine forests. *Monochamus alternatus* is the primary vector of *B. xylophilus* in East Asia. Understanding the population structure and evolutionary forces of vector insects is critical for establishing effective PWD management strategies. The present work explores the genetic structure and phylogenetic relationships of 20 populations of *M. alternatus* from the Qinling‐Daba Mountains (QDM) in China using the mitochondria DNA dataset, supplemented by ecological niche modeling (ENM). All *M. alternatus* populations were categorized into three phylogeographic clusters (Clade A, Clade B, and Clade C), with Clade A and Clade B corresponding to the western and eastern QDM, respectively. The results of divergence time estimation concur with environmental changes induced by Quaternary glacial climate oscillations in QDM of China. *M. alternatus* populations exhibited significant genetic differentiation, with expansion in their population size. Ecological niche modeling (ENM) demonstrated that precipitation and temperature significantly influence the distribution of *M. alternatus* and the species is anticipated to grow into higher latitude and higher altitude regions in the future. In a nutshell, exploring the genetic structure and evolutionary dynamics of *M. alternatus* can provide valuable insights into the prevention and occurrence of *B. xylophilus*. These findings also serve as a reference for research on population differentiation and phylogeography of other species in QDM and adjacent areas.

## Graphical abstract

The study investigates the genetic structure and phylogenetic relationships of *Monochamus alternatus* populations in China (main in Qinling‐Daba Mountains), revealing three phylogeographic clusters influenced by Quaternary glacial oscillations. Ecological niche modeling indicates that precipitation and temperature significantly affect the distribution of *M. alternatus*, suggesting future expansion to higher latitudes and altitudes.

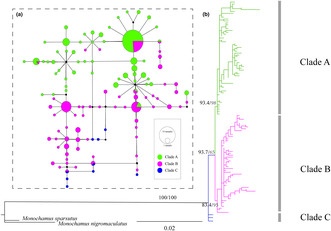

## 1 Introduction

Global climate change and trade integration increase the risk of species spread and colonization, endangering economies and ecosystems throughout the world (Millar and Stephenson 2015). Predation, competition, and symbiosis between invading and native species can significantly aid or impede invasive species establishment (DeVos, Bock, and Kolbe 2023). Vector insects play a crucial role in the spread, reproduction, and colonization of invasive species, which accelerates damage to ecosystems (Li and Zhang 2018). By defining the population origin and genetic structure of vector species, the transmission path of captured individuals can be inferred, aiding in predicting the occurrence trends of pests and risk assessment. This approach is critical for effective biological monitoring and management of high‐risk pests.

Pine wilt disease (PWD) is a deadly forest disease caused by the pine wood nematode (PWN), *Bursaphelenchus xylophilus* (Nematoda: Aphelenchoididae) (Mamiya and Enda 1972). Since its discovery in Nanjing, Jiangsu Province, China, in 1982, the PWN has spread rapidly to 663 counties in 18 provinces (and municipalities) in China (State Forestry Administration of China 2024). The PWD has destroyed billions of pine trees, incurring economic losses estimated at hundreds of billions of yuan in China (Ye 2019). PWD‐related losses in Europe are estimated at €22 billion between 2008 and 2030 (Soliman et al. 2012). In Japan, PWD causes the annual death of 1,000,000 m^3^ pine trees (Shin et al. 2009). South Korea lost roughly 7811 ha of pine trees to PWD by 2005 (Shin et al. 2009). *Monochamus alternatus* Hope (Coleoptera: Cerambycidae) is the main vector of *B. xylophilus* in Asia. *M. alternatus* adults emerging from pine trees infected with PWN can transmit PWN to healthy pine trees via feeding wounds. Dying PWN‐infected trees, on the other hand, attract *M. alternatus*, which lays eggs on them. This mutualistic interaction results in the proliferation of PWN and *M. alternatus*, causing extremely high coniferous mortality in forests (Zhao et al. 2020). As a result, effective *M. alternatus* management can significantly slow the spread of PWD. Understanding how geographical features and environmental conditions influence the distribution and genetic structure of *M. alternatus* is crucial for devising effective management strategies. The Qinling‐Daba Mountains (QDM), a region of complex topography and diverse climates, provides a unique setting to explore these factors (Huang et al. 2016).

Subtropical China experienced geological tectonic uplift in the late Miocene and Pliocene epochs, resulting in comparatively high mountain systems such as the Qinling Mountains (Ye et al. 2016). The isolation of mountain barriers caused by geological movements is the primary driver of lineage differentiation (Cheng et al. 2016). The Qinling Mountains constitute the north–south climatic boundary and are an important ecological security barrier in China. The Daba Mountains, located south of the Qinling Mountains, roughly follow a southeast‐northwest direction, separated by the Han River (Figure S1). Mountains and rivers also serve as crucial geographical barriers, resulting in distinct geographical patterns across lineages within the QDM.

Pine trees are an important component of the QDM vegetation. More than 4 million pine trees have been destroyed or fallen since the invasion of PWD into the QDM in 2009 (Announcement No.1, 2024, Shaanxi Forestry Administration). The predominance of PWD poses a severe threat to pine forest resources and the ecological security of the region. *M. alternatus* has a modest population density and presents a minimal threat to the health of pine forests in non‐endemic PWD regions. However, being the primary vector of PWN, the population density of *M. alternatus* in QDM sharply increased with the introduction of PWN, a phenomenon previously reported in Japan (Makihara 1998). Therefore, the invasion of PWD is expanding the distribution area of *M. alternatus* in the QDM. What is the dispersal route and genetic structure of *M. alternatus* populations in QDM? Have any additional populations of *M. alternatus* been introduced and colonized as a result of the PWD invasion?

In the context of the PWN invasion, understanding the population genetic structure of *M. alternatus* in QDM will aid in revealing the dispersal patterns and pathways of *M. alternatus*. This information is vital for designing effective pest prevention strategies, as it helps to identify vulnerable regions and informs targeted management efforts. This present work evaluated the genetic differentiation pattern of *M. alternatus* in QDM using phylogeographic analysis of mitochondrial DNA (mtDNA) sequences. The demographic history and gene flow among different populations were assessed to determine the potential impact of historical climatic change on population divergence. Furthermore, integrating ecological niche modeling (ENM) with genetic analysis allows us to assess the future distribution and genetic diversity of *M. alternatus* induced by climatic changes. This combination of approaches is crucial for anticipating how genetic dynamics and environmental factors together contribute on the species' spread, thereby providing a comprehensive foundation for developing pest management strategies.

## 2 Materials and Methods

### 2.1 Study Area and Sampling

**FIGURE 1:**
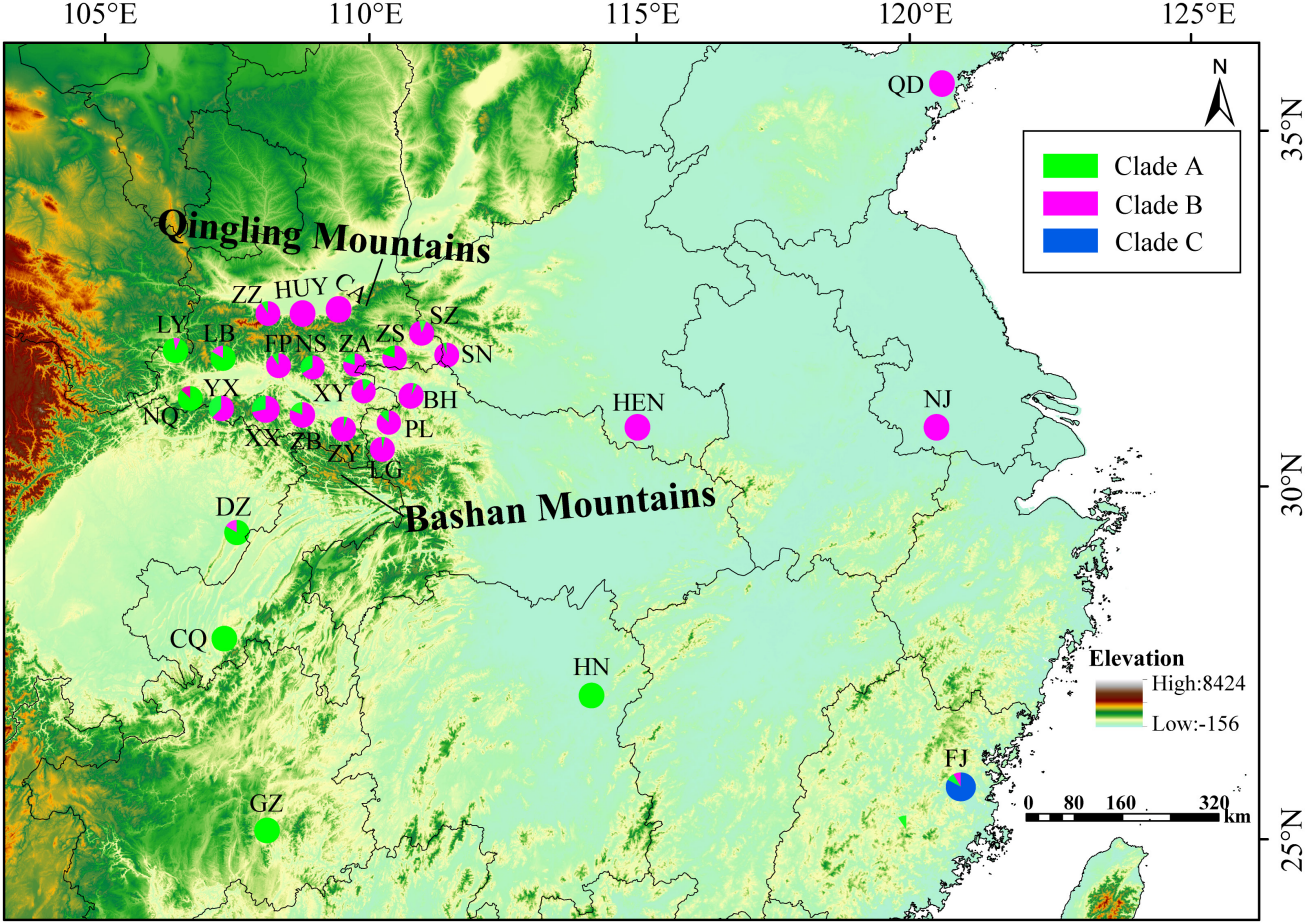
Geographic distribution of 28 populations of *Monochamus alternatus*. Pie charts for each population correspond to the proportion of each genetic cluster in the population. Clades A–C correspond to those shown in the phylogenetic tree (Figure 2). The figure was generated in ArcGIS 10.8. Table 1 provides detailed information on the location of population codes.

This study collected 186 adults of *M. alternatus* from 20 areas in QDM between 2021 and 2023 (31°41′ ~ 34°16′N, 106°29′ ~ 110°98′ E). In addition, 116 adults were collected from Henan, Sichuan, Jiangsu, Guizhou, Fujian, Hunan, Shandong, and Chongqing of China (Table 1 and Figure 1). Two species from the same genus (*M. nigromaculatus* and *M. sparsutus*) were sampled and used as outgroups in phylogenetic analysis. All insect specimens were captured in traps with the cooperation of the local forestry department (Chen Kan Agroforestry, Fujian, China; Hangzhou Pheromone Biotechnology Co., Ltd.) baited with F8‐lure (Hangzhou Pheromone Biotechnology Co., Ltd.). Collected samples were immersed in 10 mL falcon tubes containing 95% ethanol and then stored at −20°C until further processing.

**TABLE 1:** Sampling information and genetic diversity statistics of 28 geographical populations based on mtDNA combined sequences.

Mountains	Code	Population location	Latitude (N)	Longitude (E)	Number of sequences	S	*Pi*	h	*Hd*
Qinling‐Daba Mountains	CA	Changan, Shaanxi	34.1486	108.9214	4	9	0.00395	2	0.667
	HUY	Huyi, Shaanxi	34.0292	108.5251	10	4	0.00083	4	0.533
	LY	Lueyang, Shaanxi	33.5578	106.2493	10	8	0.00193	3	0.600
	FP	Foping, Shaanxi	33.3036	108.0311	14	12	0.00277	4	0.571
	LB	Liuba, Shaanxi	33.4269	106.9849	10	5	0.00124	4	0.644
	NS	Ningshan, Shaanxi	33.3140	108.3100	10	5	0.00084	3	0.511
	ZZ	Zhouzhi, Shaanxi	34.1612	108.2311	10	23	0.00533	7	0.911
	XX	Xixiang, Shaanxi	32.9563	107.8530	13	11	0.00201	6	0.821
	ZB	Zhenba, Shaanxi	32.5620	107.8836	10	10	0.00307	6	0.889
	NQ	Ningqiang, Shaanxi	32.8260	106.2950	16	17	0.00348	8	0.900
	YX	Yangxian, Shaanxi	33.2228	107.5456	5	9	0.00277	4	0.900
	PL	Pingli, Shaanxi	32.3400	109.5000	10	11	0.00211	4	0.733
	BH	Baihe, Shaanxi	32.8166	110.1190	4	14	0.00549	3	0.833
	LG	Langao, Shaanxi	32.3161	108.9069	10	9	0.00283	4	0.733
	XY	Xunyang, Shaanxi	32.8411	109.3710	7	5	0.00176	3	0.667
	ZY	Ziyang, Shaanxi	32.5114	108.5068	6	9	0.00211	5	0.933
	SZ	Shangzhou, Shaanxi	33.8561	109.9247	6	3	0.00066	2	0.333
	ZS	Zhashui, Shaanxi	33.5310	109.3750	12	12	0.00233	4	0.561
	SN	Shangnan, Shaanxi	33.5149	110.9849	9	5	0.00102	5	0.806
	ZA	Zhenan, Shaanxi	33.3318	109.1715	10	24	0.00452	7	0.867
Henan	HEN	Xinyang, Henan	33.6664	111.0576	9	18	0.00304	7	0.944
Sichuan	DZ	Dazhou, Sichuan	30.7415	107.2086	17	18	0.00284	10	0.794
Chongqing	CQ	Chongqing	30.3388	107.5471	15	23	0.00427	10	0.924
Jiangsu	NJ	Nanjing, Jiangsu	31.6663	119.3453	12	3	0.00042	4	0.561
Guizhou	GZ	Qiannan, Guizhou	26.3553	107.5146	27	10	0.00076	10	0.610
Fujian	FJ	Fuzhou, Fujian	26.1520	119.1145	9	18	0.00399	9	1.00
Hunan	HN	Changsha, Hunan	28.2442	113.0550	24	26	0.00317	15	0.953
Shandong	QD	Qingdao, Shandong	36.3902	120.4477	3	3	0.00132	2	0.667

Abbreviations: *h*, number of haplotypes; Hd, haplotype (gene) diversity; Pi, nucleotide diversity; *S*, number of polymorphic (segregating) sites.

### 2.2 DNA Extraction, Amplification, and Sequencing

Genome DNA was extracted from adult leg muscle using the SteadyPure Universal Genomic DNA Kit (Accurate Biology, AG21009, China) following the manufacturer's instructions. Three genes of cytochrome c oxidase subunit I (*COI*), cytochrome c oxidase subunit II (*COII*), and cytochrome b (*Cytb*) were amplified (refer to Table S1 for detailed primer information). The amplification reaction contained 1 μL template DNA (50 ng/μL), 1 μL each of forward and reverse primers (10 μmoL/L; Tsingke Biotechnology Co., Ltd., Xi'an, China), 5 μL 5 × Buffer (Mg^2+^plus), 2 μL dNTP mixture (2.5 mmol/L), 0.25 μL (5 U) Taq DNA polymerase (Vazyme Biotech Co., Ltd., Nanjing, China), and ddH_2_O was added to achieve 25 μL final mix.

PCR amplifications conditions: an initial denaturation at 94°C for 30 s; followed by 35 cycles of denaturation at 98°C for 10 s, annealing at 52°C (*COI* and *Cytb*) or 54°C (*COII*) for 30 s, and elongation at 72°C for 1 min; then a final elongation at 72°C for 2 min. PCR products were documented and evaluated using 1.5% agarose gel electrophoresis, and positive results were purified, and sequenced by Tsingke Biotechnology and Service Co., Ltd. (Xi'an, China). All sequences have been deposited in GenBank (Accession numbers: *COI*: PP421600‐PP421933; *COII*: PP448350‐PP448715; *Cytb*: PP442195‐PP442560; *Monochamus nigromaculatus*: *COI*: PQ146562; *COII*: PQ166617; *CYTB*: PQ155518; *Monochamus sparsutus*: *COI*: PQ146563; *COII*: PQ166618; *CYTB*: PQ155519).

### 2.3 Phylogenetic Lineage

#### 2.3.1 Genetic Diversity and Population Structure

The peak plot quality of each sequence was assessed using Chromas Pro (Technelysium Pty Ltd., Australia). All sequences were translated into amino acids to determine codon positions using Primer prime v5.0 (Premier Biosoft International, Palo Alto, CA) (Singh et al. 1998). Multiple sequence alignment was performed using Clustal X v1.81 (Thompson et al. 1997). The gappy regions at the start and end of the alignment were manually deleted with BioEdit v7.0.9.0 (Hall 1999). The substitution saturation level of each gene and the codon position of each protein‐coding gene were determined using DAMBE v3.7 (Xia 2013).

Three genes *COI*, *COII*, and *Cytb* from different populations were concatenated using PhyloSuite v.1.2.2 (Zhang et al. 2020). DNAsp v.5.0 was used to determine the number of haplotypes (*h*), nucleotide diversity (*Pi*), haplotype diversity (*Hd*), and the average number of nucleotide differences (*k*) (Librado and Rozas 2009). Arlequin v3.5 was used to evaluate molecular variance (AMOVA) and assess the differentiation index (*F*_*ST*_) between pairs of populations (Excoffier and Lischer 2010). Tajima's *D* (1989) and Fu's *Fs* (1997) were computed to perform neutrality tests. A total of 10,000 simulations were performed on each statistic to obtain a 95% confidence interval and test whether the sequence conforms to the neutral evolutionary model. The goodness of linear fitting was evaluated based on the sum of squared deviations (SSD) and Harpending's raggedness index (HRag) using Arlequin v3.5.

The correlation between the degree of genetic differentiation across different geographical populations of *M. alternatus* and geographical distance was evaluated by calculating the genetic distance *F*_*ST*_ and geographical distance (In km) between populations using GenAlEx 6.502 (Peakall and Smouse 2012). The correlation between the geographical distance and genetic distance was examined for all pairs of *M. alternatus* populations using the Mantel test with Arlequin v3.5. The Nm value computed in GenAlEx 6.502 was used to estimate gene flow based on *F*_*ST*_ (Nm = (1‐ *F*_*ST*_)/4 *F*_*ST*_).

#### 2.3.2 Haplotype Relationship Analysis

Haplotype networks were constructed using PopART v.1.7 to reveal relationships between haplotypes (Leigh and Bryant 2015). The haplotype networks were visualized and manually adjusted. A phylogenetic tree of haplotypes was reconstructed using the maximum likelihood (ML) approach. The ML analysis was performed with raxmlGUI v1.3, a graphical RAXML front‐end (Silvestro and Michalak 2012). The GTR + G models were identified as the best for phylogeny. The maximum likelihood analyses were executed 10 times using the “thorough” bootstrap setting, starting from random seeds and repeated until the likelihood score and parameter estimates stabilized. The bootstrap support value (BS) was calculated using 1000 replications. Two closely related species *M. nigromaculatus* and *M. sparsutus*, were used as outgroups. The resultant tree was edited and annotated using FigTree v1.4 (Rambaut 2006).

#### 2.3.3 Divergence Time Estimation

Divergence periods between haplotype lineages in all *M. alternatus* populations were determined using BEAST v1.8.0 (Drummond and Rambaut 2007). The GTR + G model was selected after testing an uncorrelated relaxed clock model with lognormal distribution. Insect mitochondrial *COI* genes with a mutation rate of 2.3% per million years (i.e., a substitution rate of 0.0115/locus per lineage) were selected to estimate coalescent time due to a lack of fossil calibration information for *M. alternatus* (Owen et al. 2017). We conducted two tests on 500 million generations, sampling every 10,000 generations and burning the first 25% of these runs. MCMC convergence was assessed using posterior probabilities and effective sample sizes (ESS > 200) using Tracer v1.6 (Rambaut et al. 2013). A maximum clade credibility (MCC) tree was generated with a 25% burn‐in removed using TreeAnnotator v1.8.0 (Drummond et al. 2012) and visualized in FigTree v1.4.

### 2.4 Niche Modeling and Key Environmental Variables

#### 2.4.1 Data Source

We collected 240 occurrence sites of *M. alternatus*, with the following data sources. (1) Field investigation. The majority of QDM sampling sites were obtained through field sampling, which involves utilizing GPS to record latitude and longitude. (2) Search species distribution databases including global biodiversity information facility (GBIF, http://www.gbif.org/), Center for Agriculture and Bioscience International (CABI, https://www.cabi.org/), and National Center for Biotechnology Information (NCBI, https://www.ncbi.nlm.nih.gov). SDMToolbox v1.1b was used to screen and calibrate all distribution sites to reduce the effects of spatial autocorrelation and sampling deviation (Brown 2014). Bioclimatic variables (Bio 1–19) were obtained from the WorldClim with a resolution of 2.5 min (http://www.worldclim.org/) (Fick and Hijmans 2017; Hijmans et al. 2005). The Coupled Model Comparison Program (CMIP) and the shared socioeconomic pathways (SSPs) are collaborative efforts to investigate the impact of climate change on different socioeconomic pathways. The Beijing Climate Center Climate System Model 2 Medium Resolution (BCC_CSM2_MR) was selected to predict the potential distribution regions of *M. alternatus* in the 2050s and 2070s using SSP245 and SSP585.

#### 2.4.2 Data Processing

Pearson's correlation analysis was conducted to assess the relationship between variables and multicollinearity issues (Figure S2). Environmental factors with correlation coefficients exceeding 0.8 and those with minimal contribution rates were excluded, showing the selection of bioclimatic variables with statistical and biological significance. We then selected variables that are ecologically relevant to *M. alternatus*, focusing on temperature and precipitation patterns, which are known to affect the species' survival, development, and distribution (Skendzic et al. 2021). These variables were considered to have the most substantial influence on restricting the distribution of *M. alternatus*.

To avoid or decrease overfitting in the MaxEnt model, the regularization multiplier (RM) and feature combinations (FC) were computed using the “ENMeval” package of R software to optimize the model (Kass et al. 2021). A total of 48 models were created with six feature combinations (L, LQ, H, LQH, LQHP, and LQHPT in which *L* = linear, *Q* = quadratic, *H* = hinge, *P* = product, and *T* = threshold) and eight regularization multipliers (0.5, 1.0, 1.5, 2.0, 2.5, 3.0, 3.5, and 4.0) (Phillips, Anderson, and Schapire 2006). Detailed descriptions of these features are provided as Supporting Information. The Akaike information criterion coefficient (AICc) was calculated using the “checkerboard2” method and the lowest delta AICc score was chosen to run the final MaxEnt models. As a result, the optimized model parameters for this investigation were set as FC = LQHP, beta = 1.5. The response curves and jackknife test were used to determine the relative contribution of bioclimatic variables to the model. The analysis was performed using the default program settings (random test percentage: 25%, convergence threshold: (10–5), and maximum iterations: 5000).

The accuracy of the model was tested using the area under the curve (AUC) of the receiver operating characteristic (ROC) curve (Elith, Kearney, and Phillips 2010). The AUC value varies between ranged between 0 and 1, with larger values indicating better model prediction ability. The predicted habitat suitability of *M. alternatus* was reclassified into four levels: unsuitable area (0–0.2), marginal area (0.2–0.4), moderate area (0.4–0.6), and highly‐suitable area (> 0.6). Other SDM‐related operations were implemented using the SDMToolbox v1.1b program in ArcGIS 10.8.

## 3 Results

### 3.1 Genetic Diversity and Haplotype

A total of 302 samples were evaluated from 28 populations of *M. alternatus*. The concatenated DNA fragment of three mitochondrial genes was 1568 bp (609 bp for *COI*, 529 bp for *COII*, and 430 bp for *Cytb*). Nucleotide usage showed a high A‐T bias (72.5%). There were 112 variable sites, accounting for 7.14% of the total nucleotide sequence. No indel and inframe stop codons were detected. Haplotype diversity (*Hd*) among the 28 populations of *M. alternatus* ranged between 0.88 and 1.00. The nucleotide diversity (*Pi*) ranged from 0.00366 to 0.00399 (Table 1).

The 120 haplotypes were identified from 302 samples. Among these haplotypes, 78 were singletons. Hap.10 was the predominant haplotype, shared by 74 samples, accounting for 24.50% of the total samples. Other high‐frequency haplotypes included Hap. 43 (percent: 5.63%), Hap. 23 (5.30%), Hap. 79 (2.65%), Hap. 41 (2.32%), and Hap. 66 (2.32%). The majority of distinct haplotypes were found at low frequencies (Table S2). The Mantel test detected a low but significant correlation between genetic distance and geographic distance across *M. alternatus* populations (Mantel test: *R* = 0.274, *p* < 0.0001) (Figure S3). The intraspecific divergence values *F*_*ST*_ ranged from 0.0012 (QD and HEN) to 0.8525 (QD and SZ). Similarly, gene flow (Nm) values ranged between 206.3616 (QD and HEN) and 0.0433 (QD and SZ) (Table S3).

### 3.2 Population Structure and Phylogeny

**FIGURE 2:**
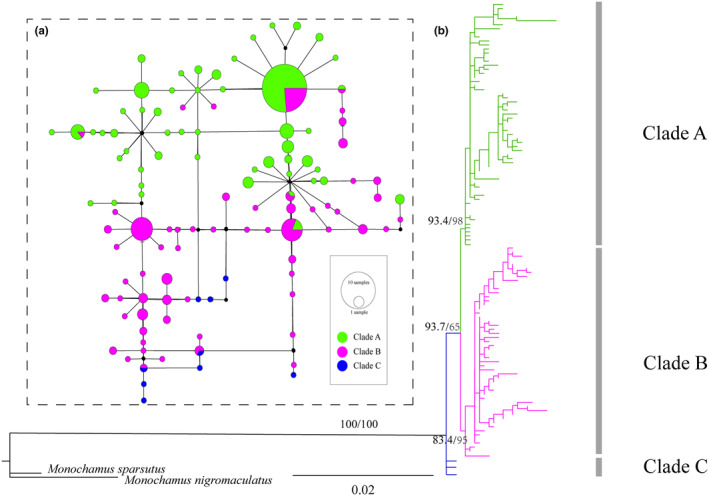
Phylogenetic tree and haplotype network profile. **(**a) Haplotype network of *Monochamus alternatus* based on mtDNA data. (b) Maximum likelihood (ML) phylogenetic tree based on the concatenated mitochondrial genes. The circle size of the haplotype denotes the number of observed individuals. Colors correspond to different regions. Numbers at the nodes are posterior probabilities.

The AMOVA analysis of *M. alternatus* geographical populations revealed that 40.98% of the total variance was within populations, while 59.02% was between populations (*F*_*ST*_ = 0.40978, *p* < 0.01) (Table 2). The ML phylogeny was derived by analyzing the concatenated mtDNA haplotypes (Figure 2). All major nodes in the ML tree have support values exceeding 70% (Hillis and Bull 1993). The phylogenetic results revealed that 28 populations of *M. alternatus* were classified into three haplogroups: Clade A, Clade B, and Clade C. The topology of the haplotype network and the evolutionary tree was congruent and presented three clades (Figure 2a,b). Haplotypes in Clade C were directly linked to outgroups and were distributed near the root of the phylogenetic tree and haplotype network. In contrast, haplotypes in Clade A and Clade B appeared near the end of the evolutionary tree and network, signifying a more derived evolutionary position (Figure 2a,b). Shared haplotypes of Clade A and Clade B were mainly derived from the haplotypes of Clade C, and the number of unique haplotypes in Clade A and Clade B populations was more than those of Clade C, with a lower nucleotide diversity (*pi*). Clade A included populations from NJ, HEN, and SD and 17 populations in the central and eastern parts of QDM. Clade B comprised populations from GZ, HN, SC, and CQ and three populations in the western part of QDM, while Clade C included only the FJ population. Notably, LY, LB, NQ, and FP shared haplotypes from both Clade A and Clade B. Overall, the findings indicate a tendency in haplotypes derived from Clade C to Clade A and Clade B. *M. alternatus* exhibited an east–west differentiation trend in QDM.

**TABLE 2:** Analysis of molecular variance (AMOVA) of geographical populations of *M. alternatus* based on the combined sequence of mtDNA.

Source of variation	d.f.	Sum of squares	Variance components	Percentage of variation	Fixation indices
Among groups	27	425.932	1.30075 *Va*	40.98	*F*_*ST*_ = 0.40978, *p* < 0.01
Within populations	274	513.349	1.87354 *Vb*	59.02	
Total	301	939.281	3.17428		

Abbreviations: *F*_*ST*_, genetic differentiation within populations; *Va*, *Vb*: number of variance components.

### 3.3 Demography Analysis

**FIGURE 3:**
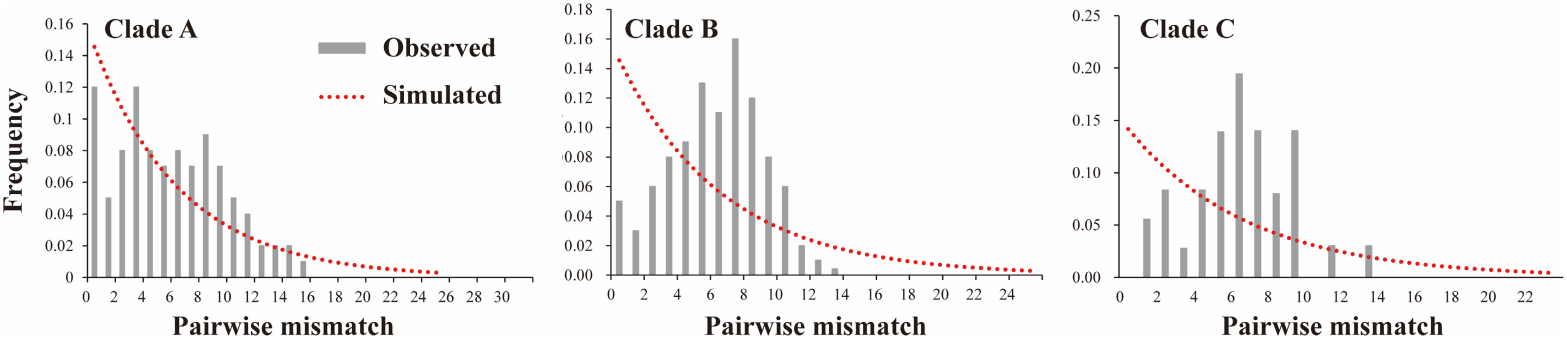
Mismatch distributions for major clades based on the mtDNA of the *Monochamus alternatus* haplotypes in each population. The bars indicate the observed values, whereas the curves represent the expected values under a population expansion model.

Mismatch analysis and neutrality tests were employed to examine historical demographic changes in *M. alternatus* using mtDNA sequences. The two curves used in this analysis represent the predicted and observed distributions, respectively. The unimodal curve, coupled with significantly negative values of Fu's *Fs* and Tajima's *D*, pointed toward recent population expansion within Clades A/B/C (*p* < 0.01). Additionally, the nonsignificant SSD and HRag values for all three clades substantiated the occurrence of the population expansion phenomenon. The three clades exhibit high haplotype diversity but low nucleotide diversity (Table 3). The mismatch distributions for lineages in Clade A, Clade B, and Clade C were unimodal, with Fu's *Fs* values of −25.100, −25.109, and −4.234 (*p* < 0.01), respectively (Table 3, Figure 3). The results of mismatch distribution and neutrality tests corroborate the conclusion of population expansion (Table 3 and Figure 3).

**TABLE 3:** The parameters of population genetic diversity in *M. alternatus*.

	NS	h	*Hd* ± SD	*Pi* ± SD	k	SSD (*p*)	Rag (*p*)	Neutrality test	
								Tajima' *D*	Fu's *F*s
Clade A	164	61	0.880 ± 0.024	0.00366 ± 0.00001	5.559	0.004 (0.950)	0.011 (1.000)	−1.741	−25.100
Clade B	129	58	0.953 ± 0.010	0.00387 ± 0.00015	5.874	0.003 (0.550)	0.012 (0.550)	−1.467	−25.109
Clade C	9	9	1.0000 ± 0.052	0.00399 ± 0.00059	6.056	0.016 (0.700)	0.048 (0.600)	−0.419	−4.234

Abbreviations: *h*, number of haplotypes; *Hd*, haplotype diversity; *k*, average number of pairwise nucleotide differences; NS, number of sequences; *Pi*, nucleotide diversity; SD, standard deviation.

### 3.4 Divergence Time Estimation

**FIGURE 4:**
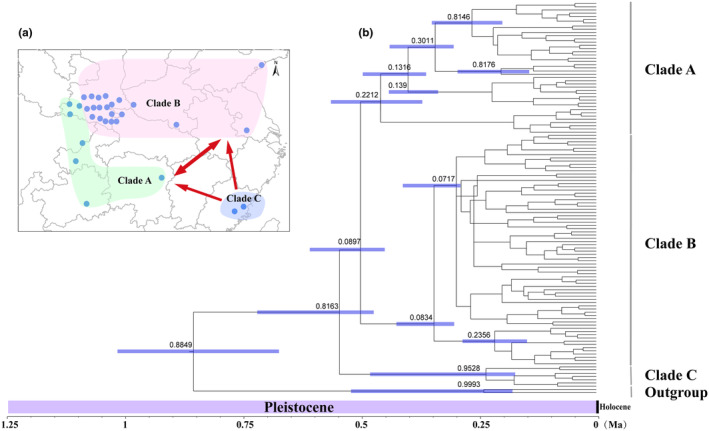
Prediction of dispersal pathways and the maximum clade credibility tree from divergence time rooted phylogenetic analysis. (a) Prediction of dispersal routes of *Monochamus alternatus* in different regions. (b) The divergence time of *Monochamus alternatus* and outgroup taxa based on *COI* gene. Estimates of divergence time are shown at nodes near branches. Divergence times at or below approximately 0.05 Ma are not shown. Purple bars and numbers under the branches provide divergence time estimates with 95% confidence ranges.

The BEAST‐derived analysis based on the concatenated mitochondrial *COI*, *COII*, and *Cytb* genes was performed on the 120 haplotypes (Figure 4). The results revealed the successive emergence of three clades (Clades A–C) over the Pleistocene. The estimated divergence period between Clade C and Clade (A + B) was ~0.82 Ma. A divergence period of ~0.09 Ma was predicted for the split of Clade A and Clade B (Figure 4b).

### 3.5 Species Distribution Model

The model was constructed using 240 screened occurrence sites of *M. alternatus* and 19 bioclimatic variables, combining FC and LQHP (Figure S4). The results demonstrated a robust prediction performance, with a test omission rate as predicted (AUC = 0.923) (Figure S5).

The maximum entropy (MaxEnt) model predicted that six specific bioclimatic variables significantly influenced the potential distribution area of *M. alternatus*, including the driest month precipitation (Bio 14), annual precipitation (Bio 12), the coldest month minimum temperature (Bio 6), the warmest quarter minimum temperature (Bio 10), mean diurnal range (mean of monthly (max temp—min temp)) (Bio 2), and mean temperature of coldest quarter (Bio 11) contributing 43.4%, 25.1%, 12.7%, 3%, 2.4%, and 2.2%, respectively. Notably, precipitation‐related bioclimatic variables (Bio 14 and Bio 12) emerged as the key determinants impacting the potential distribution of *M. alternatus*, contributing 68.5% (Table 4).

**TABLE 4:** Main topographic, bioclimatic variables and their percentage contribution used for modeling the suitable distribution of *M. alternatus*.

Abbreviation	Variables	Percentage contribution	Permutation importance
Bio_14	Precipitation of driest month/mm	43.4	0.6
Bio_12	Annual precipitation/mm	25.1	33.1
Bio_6	Min temperature of coldest month/°C	12.7	0.6
Bio_10	Min temperature of warmest quarter/°C	3	14.5
Bio_2	Mean diurnal range (mean of monthly (max temp–min temp))	2.4	1.9
Bio_11	Mean temperature of coldest quarter/°C	2.2	0.3

**FIGURE 5:**
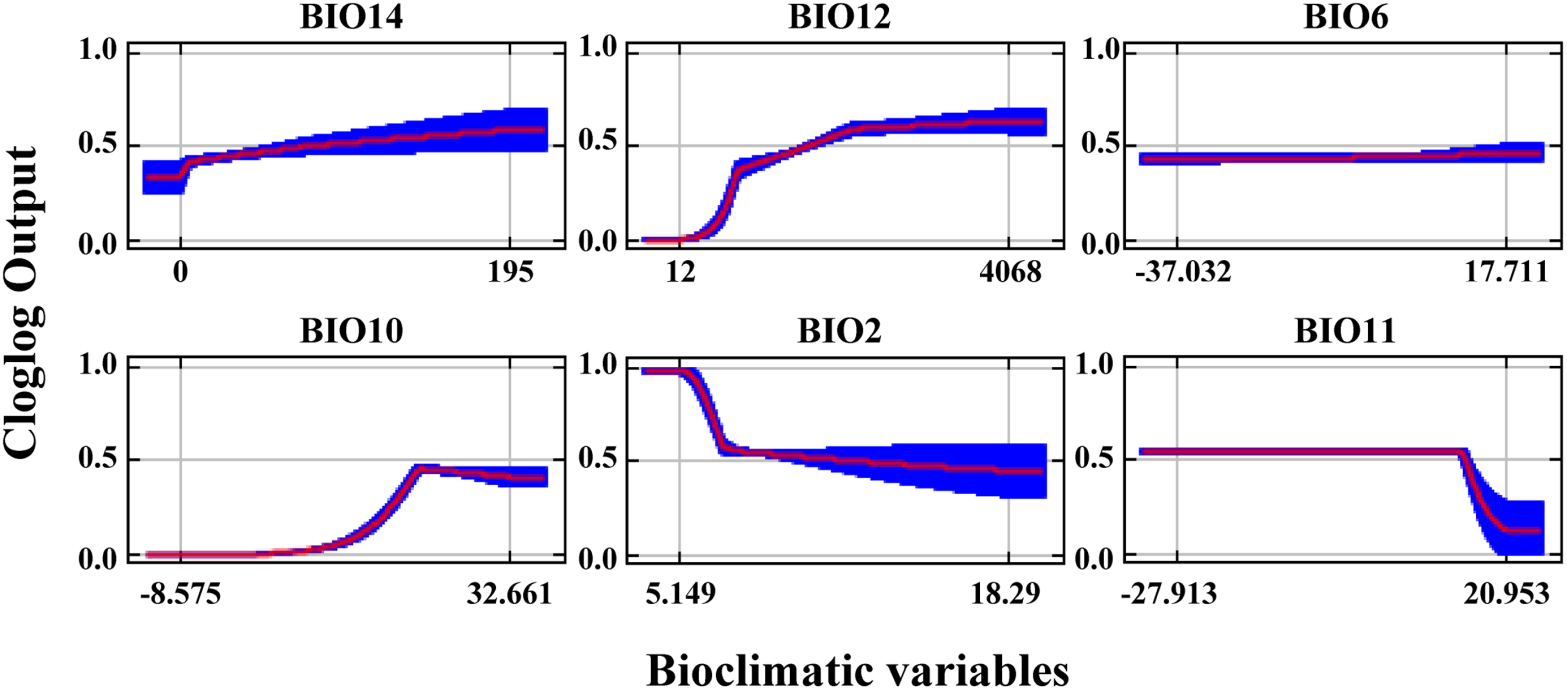
Response curves for the six bioclimatic variables obtained from the MaxEnt model.

The Jackknife test was used to determine the significance of the six selected climatic variables (Bio 14, Bio 12, Bio 6, Bio 10, Bio 2, and Bio 11) (Figure S6). Blue bands signify the relevance of the variable to species distribution, with the length of the bands positively correlated to its significance. Among these variables, Bio14 and Bio12 exerted the most considerable influence on the potential distribution of *M. alternatus*. The response curves for six key climatic parameters demonstrated a single‐peak relationship between habitat suitability and each climatic factor (Figure 5). The predicted results also establish correlations between survival probability and environmental factors. Response ranges for Bio 14, Bio 12, Bio 6, Bio 10, Bio 2, and Bio 11 were 0–195 mm, 12–4068 mm, (−37.032)‐17.711°C, (−8.575)−32.661°C, 5.149°C–18.29°C, and (−27.913)–20.953°C, respectively.

### 3.6 Prediction of Potential *M. alternatus* Distribution Areas

**FIGURE 6:**
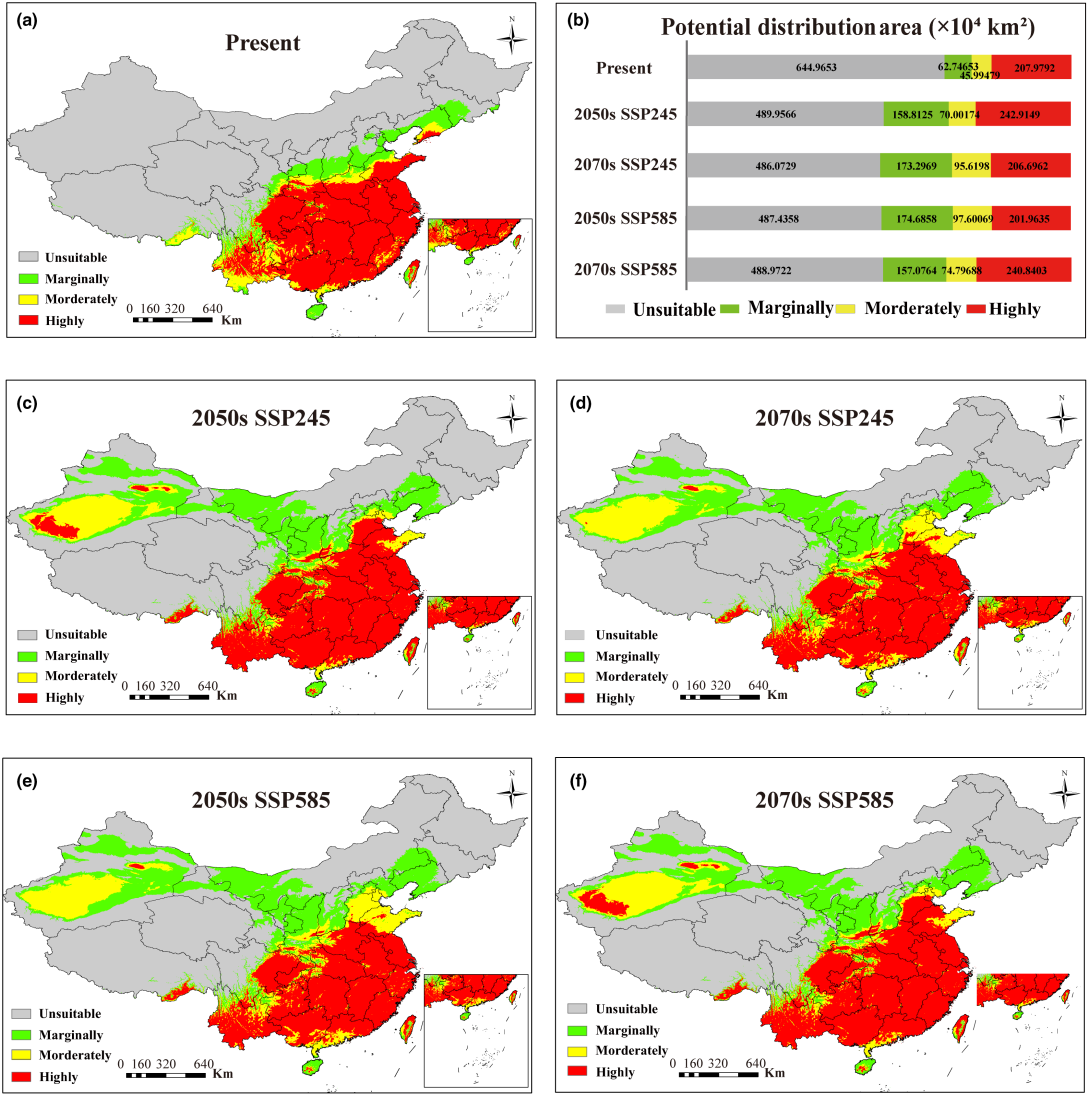
Potential distribution of the probability of occurrence for *Monochamus alternatus*. (a) Present, (b) comparison of potential distribution areas (×10^4^ km^2^), (c) 2050s SSP245, (d) 2070s SSP245, (e) 2050s SSP585, (f) 2070s SSP585. The map represents suitability as follows: gray (Unsuitable), green (Marginally suitable), yellow (Moderately suitable), and red (Highly suitable).

The potential distribution of *M. alternatus* predominantly spans southern China under the current climatic conditions (Figure 6a). The overall suitable area for *M. alternatus* in China is approximately 3,167,205.2 km^2^, comprising 19.81% marginally suitable areas (627,465.3 km^2^), 14.52% moderately suitable areas (459,947.9 km^2^), and 65.67% highly suitable area (2,079,792 km^2^) (Figure 6b).

The MaxEnt prediction for the 2050s and 2070s under two future climate scenarios SSP245 and SSP585 are shown (Figure 6c–f). In comparison to the current climate scenario, the suitable areas for *M. alternatus* in China are predicted to increase significantly under both the SSP245 and SSP585 climate scenarios. The marginally suitable areas are expected to increase by an average of 164.49% (1,032,214 km^2^), moderate habitats by an average of 83.72% (385,099.9 km^2^), and high habitats by an average of 7.27% (151,245.3 km^2^) under these two scenarios.

The new dispersal areas are primarily concentrated in northwestern China. Substantial portions of Xinjiang, Ningxia, Hebei, Liaoning, and western Inner Mongolia will transition into marginal and moderately suitable areas for *M. alternatus*. Meanwhile, Hainan will become a moderately/highly suitable area. Yunnan, Anhui, and Linzhi in Tibet are projected to transform into highly suitable areas for *M. alternatus*.

## 4 Discussion

### 4.1 Genetic Diversity and Variation

Mitochondrial genes are effective molecular markers for evaluating genetic diversity and differentiation in insect populations and they are widely used to investigate the genetic structure of insects (Fu et al. 2010; Koutroumpa et al. 2013). In the present study, three *M. alternatus* mtDNA genes, *COI*, *COII*, and *Cytb*, exhibit an evident A/T preference, which is consistent with the characteristics of insect mitochondrial genes (Huang et al. 2023). The concatenated mtDNA sequences revealed 120 haplotypes, indicating a substantial genetic diversity across *M. alternatus* populations in China. Higher *Hd* values and lower *Pi* values imply that the *M. alternatus* population may have undergone bottlenecks, followed by rapid population growth and mutation accumulation. Our results support previous studies, which have documented that the PWN and its vectors experienced rapid expansion in newly invaded areas (Li and Zhang 2018). The rapid increase in population size led to the accumulation of new mutations, although the accumulation time for nucleotide mutations remains insufficient. Meanwhile, high *Hd* values imply that *M. alternatus* has a high degree of polymorphism, adaptability, and genetic variation. The mismatch distribution analysis and negative values of neutrality tests (Tajima's *D* and Fu's *Fs*) indicated that the *M. alternatus* population had recently expanded. Genetic differentiation between populations is possible throughout the process of population expansion. AMOVA analysis revealed that the genetic variation of *M. alternatus* between different geographical populations is primarily intrapopulation (59.02% of the overall variation), with a significant genetic structure (*F*_*ST*_ = 0.4098, *p* < 0.01). This finding demonstrates that geographic isolation, habitat differences, or anthropogenic dispersal results in local adaptation or genetic drift within *M. alternatus*.

### 4.2 Biogeography

The phylogenetic tree and haplotype network show variations between the eastern and western populations of *M. alternatus* in QDM (Figure 2). Geographical barriers such as mountains or the scattered distribution of pine trees isolate and fragment *M. alternatus* habitats, restricting genetic flow among populations (Figure 1). Previous research has shown that altitude influences the distribution and dispersal of *M. alternatus* (Haran et al. 2015; Shoda‐Kagaya 2007). Limited gene flow between low‐ and high‐altitude populations can result in genetic divergence (Miño, Gardenal, and Bidau 2011). The distribution of host pine trees is also a major factor influencing the distribution of *M. alternatus*. Our genetic diversity analysis reveals that *M. alternatus* is divided into two distinct clades (Clade A and Clade B), representing the eastern and western regions of the QDM. In contrast, the distribution of pine populations in the Qinling region primarily shows a north–south pattern. For instance, *Pinus massoniana* is mainly distributed in the Daba mountains, while *P. armandii*, *P. tabuliformis*, and some *P. massoniana* are more common in the Qinling Mountains (Nan et al. 2023). All these pine species are hosts for *M. alternatus*, indicating that the distribution of pine species is not the primary factor driving the east–west population differentiation of *M. alternatus* in the QDM. Therefore, host data were not involved in the subsequent model analysis, and only focused on broad climatic factors influencing insect distribution.

Moreover, our results indicate genetic proximity between *M. alternatus* populations within QDM, as well as across numerous distantly isolated populations (i.e., DZ and HN, QD and FJ) that share haplotypes or have close genetic distances (Tables S2 and S3). These genetic similarities indicate that *M. alternatus* populations in China have undergone long‐distance exchanges. Hu et al. (2013) also reported on the long‐distance exchange between *M. alternatus* populations. Natural dispersal across larger areas can occur through the accumulation of shorter flights during an extended flight season, although the maximum flight distance of *M. alternatus* adult is only 3.3 Km (Ogawa and Hagiwara 1980). The distribution of host trees, geographical barriers such as mountains and rivers, as well as temperature and humidity, all have a significant impact on the flight range of *M. alternatus*. The observed genetic relationships between geographically distant and isolated populations, however, suggest that natural dispersal alone may not account for all gene flow. Human‐mediated movement likely played a role in facilitating these long‐distance exchanges, contributing to the genetic structure observed. In summary, our results indicate that both natural dispersal and human‐mediated migration have shaped the genetic structure of *M. alternatus* in the QDM.

### 4.3 Historical Demographics

The rapid uplift of the Qinghai–Tibet Plateau after the Pliocene influenced atmospheric circulation (Li et al. 2001). These intense movements impacted the geographical features of the eastern Qinghai–Tibetan Plateau, particularly the QDM (Li et al. 2001). The Qinling Mountains separate the Palearctic and Oriental realms and mark the transition between temperate and subtropical climatic zones (Huang et al. 2017). The Qinling Mountains and the Daba Mountains are separated by the Hanzhong Plain and the Han River (Xu et al. 2019). Previous research has revealed that species found in these mountain regions may exhibit distinct phylogeographic patterns (Huang et al. 2017). During the middle and late Pleistocene, QDM experienced drastic changes in the natural environment such as large‐scale glaciers, particularly after the Quaternary (Zhang et al. 2016). Quaternary environmental fluctuations and geographic barriers are considered major contributors to the current biodiversity of extant species (Hewitt 2004). Divergence time estimates imply that the three major clades of *M. alternatus* diverged approximately during this period (Figure 4b). The divergence of *M. alternatus* was determined to have occurred approximately 0.88 million years ago (Ma), with a subsequent divergence from populations in Fujian and other areas around 0.82 Ma. As a result, we hypothesize that *M. alternatus* population divergence was primarily influenced by climatic fluctuations during the Quaternary Ice Age. Our phylogenetic analysis and haplotype networks consistently reveal significant genetic divergence between the FJ population and others (Figure 2; Figure 3). Fujian may emerge as a pivotal refuge and origin for *M. alternatus* (Figure 4a), similar findings were reported by Hu et al. (2013). Clade A and Clade B diverged around ~0.09 Ma, indicating that the *M. alternatus* population lived during the last major ice age, known as the Late Quaternary Ice Age. The phylogeography of species was profoundly influenced by climate fluctuations during the late Miocene to late Pleistocene in the Qinling Mountains (Meng, Li, and Qiao 2014; Wang et al. 2013).

### 4.4 Prevention and Management Implications of *M. alternatus*

In recent years, PWN has steadily expanded its habitat beyond the original ecological zone, colonizing high‐altitude and high‐latitude areas in China (Li and Zhang 2018). In natural conditions, PWN is primarily transferred via vector insects, such as *Monochamus* species (Li et al. 2022). The climatic environment and human activities (including international trade) significantly influenced the spread, dispersal, and colonization of species (Du et al. 2023). As such, understanding the potential distribution and future developmental trajectory of *M. alternatus* holds crucial implications for plant quarantine and forestry management in China.

The sampling sites used to construct the model in this investigation included locations where *M. alternatus* has recently emerged, hence increasing the coverage and representativeness of the sampling sites. Modeling results indicate that bioclimatic variables related to precipitation play a more substantial role in shaping the distribution of *M. alternatus* than temperature parameters in China. Precipitation is also a major contributor to the dispersal of related species such as *Monochamus leouconotus* and *Monochamus carolinensis* (Kutywayo et al. 2013; Zhao et al. 2023). Precipitation affects insect activity both directly and indirectly (Tremblay, MacMillan, and Kharouba 2021). Precipitation can directly influence insects' feeding, mating, and reproduction by altering the moisture levels in their environment, which can either facilitate or hinder these activities. Precipitation can also indirectly affect the survival and habitat quality of insects such as *M. alternatus* by affecting the growth and distribution of host pine trees and soil microorganisms. Current evidence shows that *M. alternatus* is mainly distributed in subtropical regions of China. Insects, like poikilothermal organisms, can undergo adaptive physiological changes in response to temperature fluctuations (Guo, Sun, and Kang 2011). The distribution of *M. alternatus* has notably shifted toward warmer and middle‐temperate zones as a result of global warming. Under the SSP245 and SSP585 climate scenarios, the low‐suitability area for *M. alternatus* will increase in the 2050s and 2070s, spreading to higher latitudes and altitudes. This trend corresponds to the impact of global warming on insect distribution. Global warming is projected to cause significant temperature increases, particularly in higher latitude and altitude regions. Xiao et al. (2024) reported that under the SSP5‐8.5 scenario in 2081–2100, suitable areas of PWN will expand to higher latitudes, with significant changes occurring in suitable regions across Europe, East Asia, and North America. Like many other insect species, *M. alternatus* is ectothermic and displays a high sensitivity to fluctuations in temperature. In warmer environments, the species' metabolic rate, development speed, and reproductive capacity typically increase, allowing the species to expand its range into previously colder regions at higher latitudes and altitudes that were previously unsuitable for its survival. Xu et al. (2023) proposed that future PWD outbreaks in China are likely to follow a northward trajectory. Additionally, Zhao et al. (2023) demonstrated that temperature and precipitation exert a significant influence on species distribution.

To mitigate the spread of *M. alternatus*, rigorous quarantine measures (e.g., fumigation, microwave, and radiation treatment) for pine wood and related products are imperative. Planting disease‐resistant varieties and opting for mixed forests over monoculture plantations can be used as preventive measures as the presence of non‐hosts can slow the spread of *M. alternatus* (van Halder et al. 2022). In addition, biological control represents a sustainable solution for natural pest management. For instance, the use of parasitic wasps, such as *Dendrosoter* spp. (Manojlovic et al. 2001) and *Dastarcus helophoroides* (Li et al. 2015), or entomopathogenic fungi of *Beauveria bassiana* and *Metarhizium anisopliae* (Kim et al. 2020), illustrate the potential of these methods for the control of *M. alternatus* populations. These biological control methods can be integrated into pest management programs, thereby reducing reliance on chemical treatments. Historical collection records from QDM indicate that *M. alternatus* had a relatively limited population size before the invasion of PWN. Similar findings were reported in Japan (Kawai et al. 2006). The PWN invasion may have influenced the original population structure of *M. alternatus*, as the number of *M. alternatus* has sharply increased in PWN outbreak areas. The restricted distribution range and speed of *M. alternatus* indicate that human activities predominantly drive its dispersal (Ning et al. 2004). It is plausible that anthropogenic activities promote the hybridization of native and migratory *M. alternatus*, resulting in changes to the genetic structure of the local population. Scholarly evidence indicates that human dispersion influences the genetic pattern of *M. alternatus* in China (Shoda‐Kagaya 2007). For example, transporting PWD‐infected wood or wood packaging materials between regions causes rapid infection of pine nematodes in adjacent forests along railways or roads (Ye 2019). Moreover, Hu et al. (2013) emphasize how road traffic systems facilitated the spread of *M. alternatus* in mainland China. The dispersal trajectory of PWN closely aligns with the anthropogenic dispersal of *M. alternatus*. Consequently, the strategy for preventing and managing PWD should rigorously control disease hotspot areas and prohibit the transit of infected wood.

A population genetic analysis revealed that there is a notable differentiation in the genetic makeup of the eastern and western populations of *M. alternatus* in the QDM. This differentiation is likely influenced by geographic isolation caused by the mountain range, which has an impact on gene flow between the two populations. Furthermore, the ENM results suggest that *M. alternatus* may exhibit a tendency to expand to higher altitudes and higher latitudes in the future. This implies that currently isolated populations may potentially become more connected. Such changes in distribution and connectivity may facilitate the spread of *B. xylophilus* to new areas, particularly those that are more climatically suitable, given the effects of climate change. It is therefore imperative to gain an understanding of these dynamics in order to be able to predict future pest outbreaks and to develop proactive management strategies that take account of both the genetic resilience of *M. alternatus* and its potential for range expansion.

Overall, this study advances our understanding of genetic differentiation and the evolutionary history of *M. alternatus* in QDM, providing critical insights for the development of genetic resource management strategies. For instance, an understanding of the evolutionary history of *M. alternatus* enables the prediction of how populations will respond to environmental changes, thereby assisting in the design of long‐term management plans to mitigate the impact of this species as a vector of PWN. It should be noted that our study focused exclusively on mitochondrial genes, which may not provide a comprehensive view of genetic structure. In future research, we plan to complement our findings by incorporating additional molecular markers and expanding sample collection from a broader geographic range, aiming to strengthen the conclusions drawn in this work and expand the understanding of the evolutionary history of *M. alternatus*.

## Author Contributions

**Jingyu Qi:** data curation (equal), investigation (equal), writing – original draft (equal). **Junke Nan:** data curation (equal), investigation (equal). **Xiaogu Zhao:** data curation (equal), investigation (equal). **Chaoqiong Liang:** data curation (equal), investigation (equal). **Jiangbin Fan:** funding acquisition (equal), supervision (equal). **Hong He:** conceptualization (equal), funding acquisition (equal), methodology (equal).

## Conflicts of Interest

The authors declare no conflicts of interest.

## Supporting information


Data S1.

## Data Availability

The sequencing data were uploaded to GenBank (Accession numbers: *M. alternatus*: *COI*: PP421600‐PP421933; *COII*: PP448350‐PP448715; *Cytb*: PP442195‐PP442560; *Monochamus nigromaculatus*: *COI*: PQ146562; *COII*: PQ166617; *CYTB*: PQ155518; *Monochamus sparsutus*: *COI*: PQ146563; *COII*: PQ166618; *CYTB*: PQ155519).
