# Supplementary material for: Genetic Structure of Monochamus alternatus (Hope) in Qinling‐Daba Mountains and Expansion Trend: Implications for Pest Prevention and Management
Source: Ecol Evol. 2024 Oct 8;14(10):e70373. doi: 10.1002/ece3.70373 (PMC11461022; doi:10.1002/ece3.70373)
Supplement: Supplementary file 1 — Data S1. [file ECE3-14-e70373-s001.docx]

**Supplementary file**

**Table S1** Primers used for PCR amplification and sequencing in this study.

| Gene fragment | Primers | Sequences | Size | References |
| --- | --- | --- | --- | --- |
| *COI* | LCO1490 | 5′-GGTCAACAAATCATAAAGATATTGG-3′ | < 700 bp | (Folmer et al. 1994) |
|  | HCO2198 | 5′-TAAACTTCAGGGTGACCAAAAAATCA-3′ |  |  |
| *COII* | *COII*_F | 5′-TGCTTCAAGATAGAGCCTCTCC-3′ | < 650 bp | (Correa et al. 2017) |
|  | *COII*_R | 5′-GGTTTGCTC CACAGATTTCAG-3′ |  |  |
| *Cytb* | *Cytb*_F | 5′-TATGTACTACCA TGAGGACAAATATC-3′ | < 600 bp | (Mao et al. 2010) |
|  | *Cytb*_R | 5′-ATTACACCTCCTAATTTATTAGGAAT -3′ |  |  |

**Table S2** Haplotypes distribution of *Monochamus alternatus* in each population

| Haplotypes | Numbers of individuals | *M. alternatus* individuals and populations |
| --- | --- | --- |
| Hap_1 | 1 | [BH1] |
| Hap_2 | 1 | [HEN1] |
| Hap_3 | 1 | [HEN10] |
| Hap_4 | 1 | [HEN2] |
| Hap_5 | 1 | [HEN3] |
| Hap_6 | 1 | [HEN4] |
| Hap_7 | 3 | [HEN5 HEN6 DZ20] |
| Hap_8 | 2 | [HEN8 HEN9] |
| Hap_9 | 1 | [BH10] |
| Hap_10 | 74 | [BH7 BH9 FP1 FP16 FP17 FP18 FP3 FP5 FP7 FP8 FP9 LB14 LB16 LB18 LB19 LB21 LB9 LG17 LG18 LG19 LY1 LY10 LY12 LY13 LY17 LY20 NQ14 NQ15 NQ19 NQ63 NS12 NS13 NS14 NS15 NS16 NS2 NS22 PL12 PL13 PL16 PL17 SN1 SZ1 SZ2 SZ3 SZ5 SZ6 XX1 XX2 XXM4 XXM5 XXM9 XY2 XY5 XY6 XY8 YX6 ZA10 ZA12 ZA18 ZA2 ZB20 ZSH4 ZSH5 ZSH6 ZSY1 ZSY4 ZSY5 ZSY8 ZSY9 ZY3 ZY4 ZZ11 ZZ5] |
| Hap_11 | 2 | [CA1 CA4] |
| Hap_12 | 2 | [CA7 CA9] |
| Hap_13 | 2 | [CQ1 CQ10] |
| Hap_14 | 4 | [CQ11 CQ17 CQ4 CQ8] |
| Hap_15 | 1 | [CQ12] |
| Hap_16 | 1 | [CQ14] |
| Hap_17 | 2 | [CQ15 CQ5] |
| Hap_18 | 1 | [CQ19] |
| Hap_19 | 1 | [CQ20] |
| Hap_20 | 1 | [CQ21] |
| Hap_21 | 1 | [CQ3] |
| Hap_22 | 1 | [CQ6] |
| Hap_23 | 16 | [DZ1 DZ10 DZ18 DZ2 DZ23 DZ3 DZ7 DZ8 FP11 FP19 FP20 NQ17 NQ8 NQ9 ZB12 ZB2] |
| Hap_24 | 1 | [DZ11] |
| Hap_25 | 3 | [DZ15 HN15 HN23] |
| Hap_26 | 1 | [DZ16] |
| Hap_27 | 1 | [DZ21] |
| Hap_28 | 1 | [DZ4] |
| Hap_29 | 1 | [DZ5] |
| Hap_30 | 1 | [DZ6] |
| Hap_31 | 3 | [DZ9 ZB18 ZB19] |
| Hap_32 | 3 | [FJ10 QD2 QD4] |
| Hap_33 | 1 | [FJ11] |
| Hap_34 | 1 | [FJ2] |
| Hap_35 | 1 | [FJ3] |
| Hap_36 | 1 | [FJ4] |
| Hap_37 | 1 | [FJ5] |
| Hap_38 | 1 | [FJ7] |
| Hap_39 | 2 | [FJ8 HN18] |
| Hap_40 | 1 | [FJ9] |
| Hap_41 | 7 | [FP21 NQ6 YX1 YX2 ZZ13 ZZ3 ZZ4] |
| Hap_42 | 2 | [FP4 PL11] |
| Hap_43 | 17 | [GZ1 GZ10 GZ11 GZ14 GZ15 GZ17 GZ2 GZ22 GZ24 GZ25 GZ26 GZ27 GZ3 GZ5 GZ7 GZ8 GZ9] |
| Hap_44 | 1 | [GZ12] |
| Hap_45 | 1 | [GZ13] |
| Hap_46 | 2 | [GZ16 GZ6] |
| Hap_47 | 1 | [GZ19] |
| Hap_48 | 1 | [GZ20] |
| Hap_49 | 1 | [GZ21] |
| Hap_50 | 1 | [GZ23] |
| Hap_51 | 1 | [GZ28] |
| Hap_52 | 1 | [GZ4] |
| Hap_53 | 3 | [HN1 HN25 HN4] |
| Hap_54 | 1 | [HN11] |
| Hap_55 | 2 | [HN12 HN16] |
| Hap_56 | 4 | [HN13 HN22 HN26 HN30] |
| Hap_57 | 2 | [HN14 HN2] |
| Hap_58 | 1 | [HN17] |
| Hap_59 | 1 | [HN19] |
| Hap_60 | 1 | [HN20] |
| Hap_61 | 1 | [HN27] |
| Hap_62 | 1 | [HN29] |
| Hap_63 | 1 | [HN6] |
| Hap_64 | 1 | [HN7] |
| Hap_65 | 2 | [HN8 HN9] |
| Hap_66 | 7 | [HUY10 HUY12 HUY13 HUY14 HUY16 HUY17 HUY19] |
| Hap_67 | 1 | [HUY11] |
| Hap_68 | 1 | [HUY15] |
| Hap_69 | 1 | [HUY18] |
| Hap_70 | 1 | [LB12] |
| Hap_71 | 3 | [LB13 LB17 PL15] |
| Hap_72 | 1 | [LB15] |
| Hap_73 | 2 | [LG1 LG12] |
| Hap_74 | 1 | [LG11] |
| Hap_75 | 4 | [LG13 LG14 LG15 LG16] |
| Hap_76 | 3 | [LY11 LY2 LY21] |
| Hap_77 | 2 | [LY15 NQ1] |
| Hap_78 | 2 | [NJ1 NJ8] |
| Hap_79 | 8 | [NJ10 NJ11 NJ2 NJ3 NJ4 NJ5 NJ7 NJ9] |
| Hap_80 | 1 | [NJ12] |
| Hap_81 | 1 | [NJ6] |
| Hap_82 | 2 | [NQ10 NQ11] |
| Hap_83 | 2 | [NQ12 NQ58] |
| Hap_84 | 2 | [NQ13 NQ60] |
| Hap_85 | 2 | [NQ18 XXM11] |
| Hap_86 | 1 | [NS17] |
| Hap_87 | 2 | [NS20 NS21] |
| Hap_88 | 4 | [PL1 PL10 PL14 PL18] |
| Hap_89 | 1 | [QD3] |
| Hap_90 | 2 | [SN10 SN8] |
| Hap_91 | 5 | [SN3 SN4 SN5 SN7 ZY1] |
| Hap_92 | 1 | [SN6] |
| Hap_93 | 2 | [SN9 ZY2] |
| Hap_94 | 1 | [SZ4] |
| Hap_95 | 3 | [XXB1 XXB3 ZY6] |
| Hap_96 | 1 | [XXB4] |
| Hap_97 | 3 | [XXB5 XXM10 XXM3] |
| Hap_98 | 1 | [XXB6] |
| Hap_99 | 2 | [XY3 XY9] |
| Hap_100 | 1 | [XY7] |
| Hap_101 | 1 | [YX3] |
| Hap_102 | 1 | [YX4] |
| Hap_103 | 1 | [ZA11] |
| Hap_104 | 1 | [ZA13] |
| Hap_105 | 1 | [ZA15] |
| Hap_106 | 1 | [ZA20] |
| Hap_107 | 1 | [ZA21] |
| Hap_108 | 1 | [ZA22] |
| Hap_109 | 3 | [ZB1 ZB14 ZB16] |
| Hap_110 | 1 | [ZB11] |
| Hap_111 | 1 | [ZB13] |
| Hap_112 | 2 | [ZSH1 ZSY3] |
| Hap_113 | 1 | [ZSH2] |
| Hap_114 | 1 | [ZSY7] |
| Hap_115 | 1 | [ZY5] |
| Hap_116 | 1 | [ZZ1] |
| Hap_117 | 1 | [ZZ15] |
| Hap_118 | 1 | [ZZ16] |
| Hap_119 | 1 | [ZZ19] |
| Hap_120 | 1 | [ZZ6] |

| **Table S3** Population pairwise *F_ST_* values (black font) and gene flow *Nm* (blue font) among *Monochamus alternatus* populations |  | HEN | BH | CA | CQ | DZ | FJ | FP | GZ | HN | HUY | LB | LG | LY | NJ | NQ | NS | PL | QD | SN | SZ | XX | XY | YX | ZA | ZB | ZS | ZY | ZZ |
| --- | --- | --- | --- | --- | --- | --- | --- | --- | --- | --- | --- | --- | --- | --- | --- | --- | --- | --- | --- | --- | --- | --- | --- | --- | --- | --- | --- | --- | --- |
|  | HEN | 0 | 0.7772 | 0.3781 | 0.9066 | 0.3408 | 1.9636 | 0.3048 | 0.3061 | 1.6034 | 0.1558 | 0.1410 | 0.1367 | 0.1726 | 0.1655 | 0.2809 | 0.1149 | 0.1707 | 206.3616 | 0.1191 | 0.1312 | 0.1879 | 0.1435 | 0.5664 | 0.3991 | 0.2730 | 0.2473 | 0.1942 | 1.2887 |
|  | BH | 0.2434 | 0 | 3.0714 | 1.1329 | 0.4431 | 1.4581 | 2.7741 | 0.1844 | 0.7200 | 0.4530 | 0.5426 | 0.3298 | 0.7113 | 0.2927 | 0.8946 | 0.3970 | 0.7470 | 0.9560 | 0.3904 | 0.5590 | 0.8997 | 0.5190 | -30.7378 | -58.6612 | 0.7166 | 2.5033 | 1.1233 | -8.4467 |
|  | CA | 0.3981 | 0.0753 | 0 | 0.7465 | 0.3366 | 0.6724 | 0.7934 | 0.1388 | 0.3691 | 0.2226 | 0.2816 | 0.2361 | 0.4238 | 0.1534 | 0.6025 | 0.2167 | 0.3711 | 0.3028 | 0.2262 | 0.2629 | 0.4695 | 0.2931 | 1.1821 | 1.1111 | 0.5051 | 0.5951 | 0.4834 | 1.4712 |
|  | CQ | 0.2162 | 0.1808 | 0.2509 | 0 | 1.1242 | 1.6673 | 0.7119 | 0.8551 | 1.4328 | 0.4145 | 0.3655 | 0.2367 | 0.4243 | 0.2984 | 0.6347 | 0.2864 | 0.3333 | 1.0338 | 0.2795 | 0.3316 | 0.4688 | 0.3098 | 0.6151 | 0.6642 | 0.8632 | 0.4852 | 0.5068 | 0.8801 |
|  | DZ | 0.4231 | 0.3607 | 0.4262 | 0.1819 | 0 | 0.5191 | 0.5792 | 0.2392 | 0.4071 | 0.2606 | 0.2602 | 0.2018 | 0.3395 | 0.1858 | 1.2601 | 0.1809 | 0.2199 | 0.3611 | 0.1817 | 0.2044 | 0.3269 | 0.2141 | 0.2797 | 0.4159 | 2.7577 | 0.2961 | 0.3257 | 0.4258 |
|  | FJ | 0.1129 | 0.1464 | 0.2710 | 0.1304 | 0.3251 | 0 | 0.5560 | 0.5267 | 1.6850 | 0.2429 | 0.2580 | 0.2257 | 0.3133 | 0.2309 | 0.4487 | 0.2089 | 0.2832 | 1.6189 | 0.2255 | 0.2542 | 0.3642 | 0.2709 | 0.7177 | 0.7185 | 0.5338 | 0.4408 | 0.3949 | 1.3666 |
|  | FP | 0.4506 | 0.0827 | 0.2396 | 0.2599 | 0.3015 | 0.3102 | 0 | 0.1981 | 0.3677 | 0.7870 | 5.9596 | 0.4139 | 6.3848 | 0.3589 | 3.0078 | 1.4724 | 1.6565 | 0.2486 | 0.9991 | 2.6536 | 5.5802 | 1.1375 | 0.6797 | 12.2876 | 1.8629 | 6.2520 | 16.2082 | 0.7134 |
|  | GZ | 0.4495 | 0.5755 | 0.6431 | 0.2262 | 0.5110 | 0.3219 | 0.5579 | 0 | 0.7956 | 0.0956 | 0.0784 | 0.0707 | 0.0980 | 0.0623 | 0.1903 | 0.0614 | 0.0883 | 0.1221 | 0.0610 | 0.0580 | 0.1234 | 0.0663 | 0.1323 | 0.2060 | 0.1661 | 0.1416 | 0.0909 | 0.3138 |
|  | HN | 0.1349 | 0.2577 | 0.4038 | 0.1486 | 0.3805 | 0.1292 | 0.4047 | 0.2391 | 0 | 0.2661 | 0.2103 | 0.1576 | 0.2307 | 0.2835 | 0.3129 | 0.1740 | 0.2122 | 1.1579 | 0.1764 | 0.1960 | 0.2585 | 0.1851 | 0.5695 | 0.3923 | 0.3296 | 0.3110 | 0.2565 | 0.7253 |
|  | HUY | 0.6161 | 0.3556 | 0.5290 | 0.3762 | 0.4896 | 0.5072 | 0.2411 | 0.7234 | 0.4844 | 0 | 0.3029 | 0.1727 | 0.2917 | 0.0745 | 0.4914 | 0.1559 | 0.2868 | 0.0637 | 0.1395 | 0.1424 | 0.3051 | 0.1741 | 0.2115 | 0.7861 | 0.2928 | 0.3697 | 0.2715 | 0.4144 |
|  | LB | 0.6394 | 0.3154 | 0.4703 | 0.4062 | 0.4900 | 0.4921 | 0.0403 | 0.7614 | 0.5431 | 0.4522 | 0 | 0.2980 | 4.6771 | 0.1133 | 0.9804 | 1.6933 | 1.5308 | 0.0742 | 0.7241 | 3.1733 | 2.5880 | 0.9436 | 0.2181 | 3.6212 | 0.5762 | 2.1405 | -19.0329 | 0.3397 |
|  | LG | 0.6464 | 0.4312 | 0.5143 | 0.5136 | 0.5534 | 0.5255 | 0.3766 | 0.7796 | 0.6133 | 0.5915 | 0.4562 | 0 | 0.3460 | 0.1163 | 0.4152 | 0.2510 | 0.6344 | 0.1144 | 0.2654 | 0.2951 | 0.3081 | 0.7678 | 0.1969 | 0.7824 | 0.3196 | 0.3360 | 0.3679 | 0.2600 |
|  | LY | 0.5916 | 0.2601 | 0.3710 | 0.3707 | 0.4241 | 0.4438 | 0.0377 | 0.7183 | 0.5201 | 0.4615 | 0.0507 | 0.4194 | 0 | 0.2035 | 1.9712 | 1.2895 | 1.1386 | 0.1162 | 0.8126 | 2.1217 | 5.1836 | 1.0398 | 0.3030 | 3.2237 | 0.9314 | 2.7519 | 6.9133 | 0.3904 |
|  | NJ | 0.6017 | 0.4607 | 0.6198 | 0.4559 | 0.5737 | 0.5198 | 0.4106 | 0.8006 | 0.4686 | 0.7704 | 0.6882 | 0.6826 | 0.5513 | 0 | 0.3668 | 0.0841 | 0.1550 | 0.0467 | 0.0830 | 0.0655 | 0.2106 | 0.0984 | 0.2328 | 0.4223 | 0.2041 | 0.3378 | 0.1333 | 0.3341 |
|  | NQ | 0.4709 | 0.2184 | 0.2933 | 0.2826 | 0.1656 | 0.3578 | 0.0767 | 0.5678 | 0.4441 | 0.3372 | 0.2032 | 0.3759 | 0.1126 | 0.4053 | 0 | 0.5489 | 0.6067 | 0.2817 | 0.5054 | 0.7025 | 1.1639 | 0.6211 | 0.4946 | 1.2711 | 7.1993 | 0.9150 | 1.1923 | 0.4940 |
|  | NS | 0.6851 | 0.3864 | 0.5357 | 0.4660 | 0.5802 | 0.5447 | 0.1452 | 0.8027 | 0.5897 | 0.6159 | 0.1287 | 0.4990 | 0.1624 | 0.7484 | 0.3129 | 0 | 1.1818 | 0.0509 | 0.6951 | 4.3062 | 1.5096 | 0.7487 | 0.1609 | 1.8651 | 0.3531 | 1.6607 | 2.3479 | 0.2801 |
|  | PL | 0.5942 | 0.2508 | 0.4025 | 0.4286 | 0.5321 | 0.4689 | 0.1311 | 0.7390 | 0.5409 | 0.4657 | 0.1404 | 0.2827 | 0.1800 | 0.6173 | 0.2918 | 0.1746 | 0 | 0.1133 | 0.7266 | 1.8778 | 1.2066 | 3.3052 | 0.3094 | 7.5503 | 0.4263 | 1.5893 | 2.0000 | 0.3903 |
|  | QD | 0.0012 | 0.2073 | 0.4523 | 0.1947 | 0.4091 | 0.1338 | 0.5015 | 0.6719 | 0.1776 | 0.7969 | 0.7712 | 0.6861 | 0.6828 | 0.8427 | 0.4702 | 0.8307 | 0.6882 | 0 | 0.0540 | 0.0433 | 0.1323 | 0.0844 | 0.3520 | 0.4027 | 0.2454 | 0.1724 | 0.1189 | 2.2361 |
|  | SN | 0.6773 | 0.3904 | 0.5250 | 0.4722 | 0.5791 | 0.5258 | 0.2002 | 0.8038 | 0.5862 | 0.6418 | 0.2567 | 0.4851 | 0.2353 | 0.7509 | 0.3310 | 0.2645 | 0.2560 | 0.8223 | 0 | 0.7841 | 0.9396 | 0.5905 | 0.1649 | 1.7182 | 0.3426 | 1.0228 | 13.0904 | 0.2919 |
|  | SZ | 0.6559 | 0.3090 | 0.4875 | 0.4299 | 0.5501 | 0.4958 | 0.0861 | 0.8118 | 0.5606 | 0.6372 | 0.0730 | 0.4586 | 0.1054 | 0.7925 | 0.2625 | 0.0549 | 0.1175 | 0.8525 | 0.2418 | 0 | 2.7437 | 0.9504 | 0.1803 | 4.6385 | 0.4336 | 3.3219 | 7.8748 | 0.3560 |
|  | XX | 0.5709 | 0.2175 | 0.3475 | 0.3478 | 0.4333 | 0.4070 | 0.0429 | 0.6695 | 0.4916 | 0.4503 | 0.0881 | 0.4480 | 0.0460 | 0.5427 | 0.1768 | 0.1421 | 0.1716 | 0.6540 | 0.2102 | 0.0835 | 0 | 0.9835 | 0.3225 | 2.4437 | 0.8516 | 3.2073 | -11.4961 | 0.4053 |
|  | XY | 0.6353 | 0.3251 | 0.4603 | 0.4466 | 0.5386 | 0.4799 | 0.1802 | 0.7904 | 0.5746 | 0.5894 | 0.2095 | 0.2456 | 0.1938 | 0.7175 | 0.2870 | 0.2503 | 0.0703 | 0.7476 | 0.2974 | 0.2083 | 0.2027 | 0 | 0.2118 | 4.0140 | 0.4390 | 0.9897 | 1.4483 | 0.3376 |
|  | YX | 0.3062 | -0.0082 | 0.1746 | 0.2890 | 0.4720 | 0.2584 | 0.2689 | 0.6540 | 0.3051 | 0.5418 | 0.5341 | 0.5594 | 0.4521 | 0.5178 | 0.3357 | 0.6085 | 0.4469 | 0.4153 | 0.6025 | 0.5810 | 0.4367 | 0.5414 | 0 | 1.0331 | 0.3384 | 0.5171 | 0.3122 | 13.6621 |
|  | ZA | 0.3852 | -0.0043 | 0.1837 | 0.2735 | 0.3754 | 0.2581 | 0.0199 | 0.5482 | 0.3892 | 0.2413 | 0.0646 | 0.2422 | 0.0720 | 0.3718 | 0.1644 | 0.1182 | 0.0321 | 0.3831 | 0.1270 | 0.0511 | 0.0928 | 0.0586 | 0.1948 | 0 | 0.8936 | -18.1583 | 10.2366 | 0.9726 |
|  | ZB | 0.4780 | 0.2586 | 0.3311 | 0.2246 | 0.0831 | 0.3189 | 0.1183 | 0.6009 | 0.4313 | 0.4606 | 0.3026 | 0.4389 | 0.2116 | 0.5505 | 0.0336 | 0.4145 | 0.3697 | 0.5047 | 0.4219 | 0.3657 | 0.2270 | 0.3628 | 0.4249 | 0.2186 | 0 | 0.6270 | 0.9295 | 0.4742 |
|  | ZS | 0.5027 | 0.0908 | 0.2958 | 0.3401 | 0.4578 | 0.3619 | 0.0385 | 0.6384 | 0.4456 | 0.4034 | 0.1046 | 0.4266 | 0.0833 | 0.4253 | 0.2146 | 0.1308 | 0.1359 | 0.5919 | 0.1964 | 0.0700 | 0.0723 | 0.2017 | 0.3259 | -0.0140 | 0.2851 | 0 | 4.2087 | 0.5904 |
|  | ZY | 0.5628 | 0.1820 | 0.3409 | 0.3304 | 0.4342 | 0.3877 | 0.0152 | 0.7333 | 0.4936 | 0.4794 | -0.0133 | 0.4046 | 0.0349 | 0.6522 | 0.1733 | 0.0962 | 0.1111 | 0.6777 | 0.0187 | 0.0308 | -0.0222 | 0.1472 | 0.4447 | 0.0238 | 0.2120 | 0.0561 | 0 | 0.4737 |
|  | ZZ | 0.1625 | -0.0305 | 0.1453 | 0.2212 | 0.3700 | 0.1547 | 0.2595 | 0.4434 | 0.2563 | 0.3763 | 0.4239 | 0.4902 | 0.3904 | 0.4280 | 0.3360 | 0.4716 | 0.3905 | 0.1006 | 0.4614 | 0.4125 | 0.3815 | 0.4255 | 0.0180 | 0.2045 | 0.3452 | 0.2975 | 0.3455 | 0 |


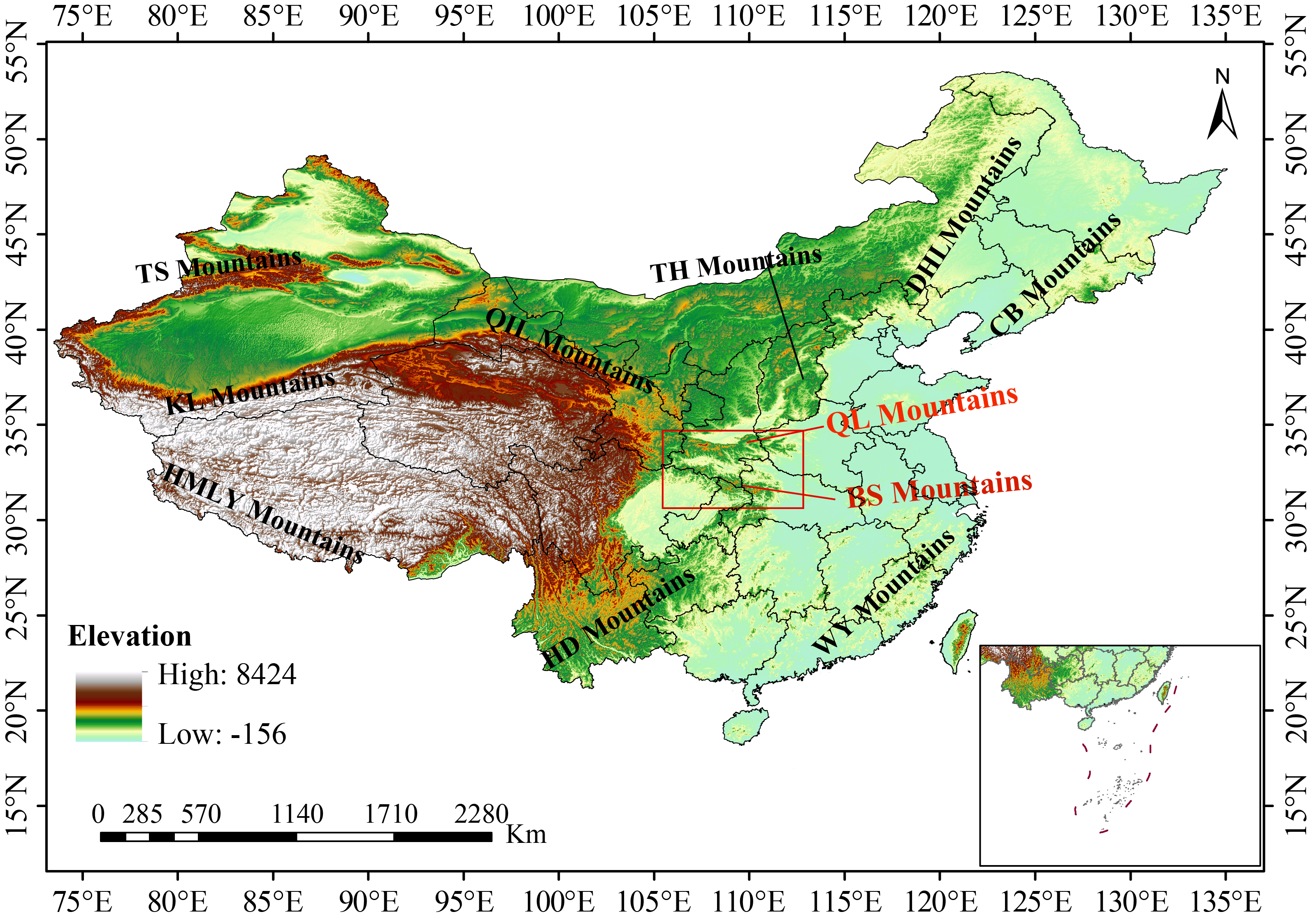


**Figure S1** Map of the general distribution of the Qinling-Daba Mountains in China. DHL: DaHingganLing; CB: ChangBai; QL: QinLing; BS: DaBa; TH: TaiHang; WY: WuYi; QIL: QiLian; HD: HengDuan; TS: TianShan; KL: KunLun; HMLY: Himalaya.


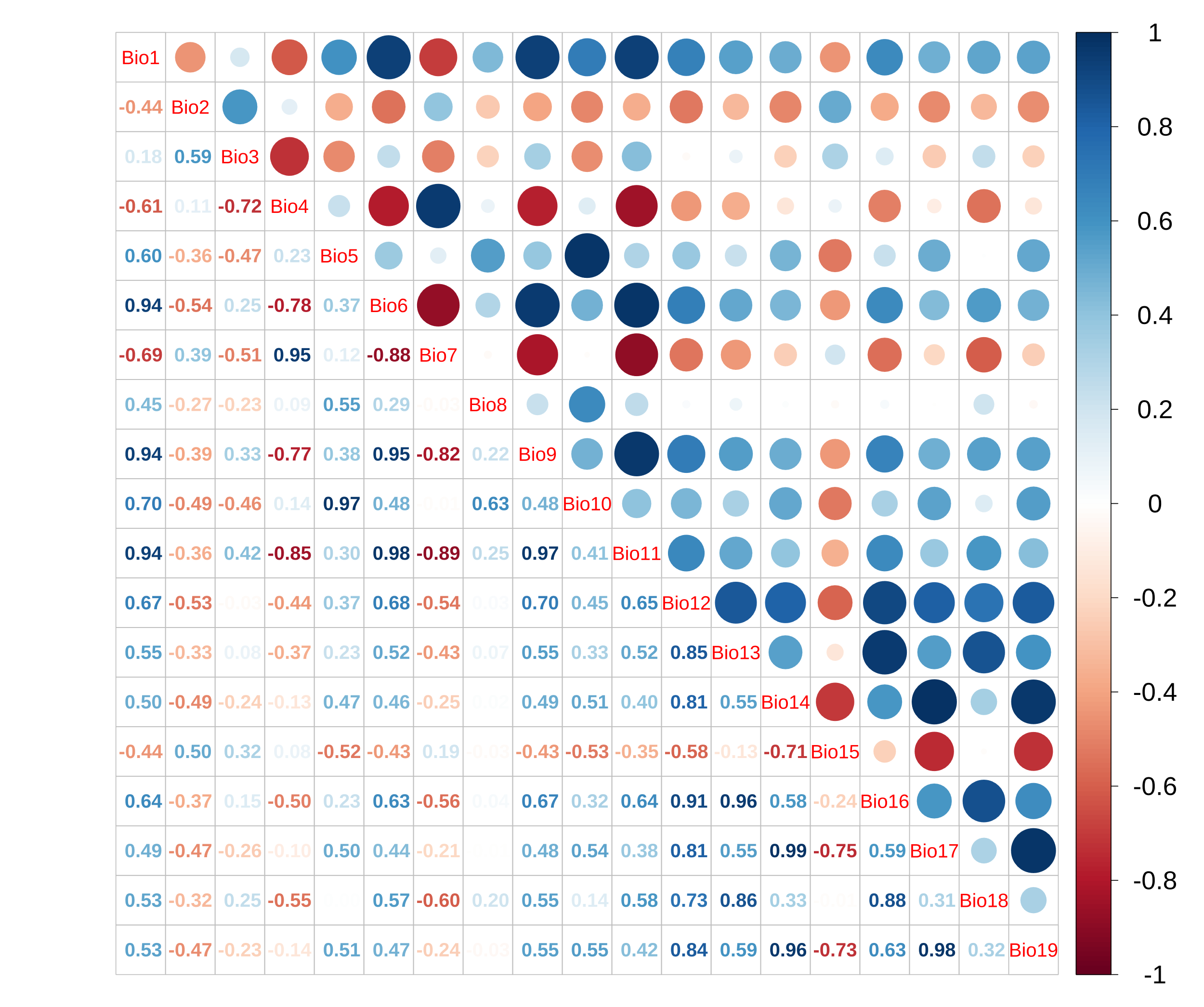


**Figure S2** Correlation heatmap of 19 environmental variables

Red represented positive correlation, blue represented negative correlation, the deeper the color, the higher the correlation.

**Figure S3** Scatter plots of genetic distance vs. geographical distance for pairwise population comparisons (10000 iterations)

**Figure S****4** The performance the of ecological niche model of *M. alternatus* under different settings. The optimal parameters are FC is LQH and RM is 1.5. Six FC combinations, β multiplier settings from 0.5 to 4, 0.5 increments. β: Regularization multiplier; FC: feature combination, L-linear, Q-quadratic, H-hinge, P-product, T-threshold


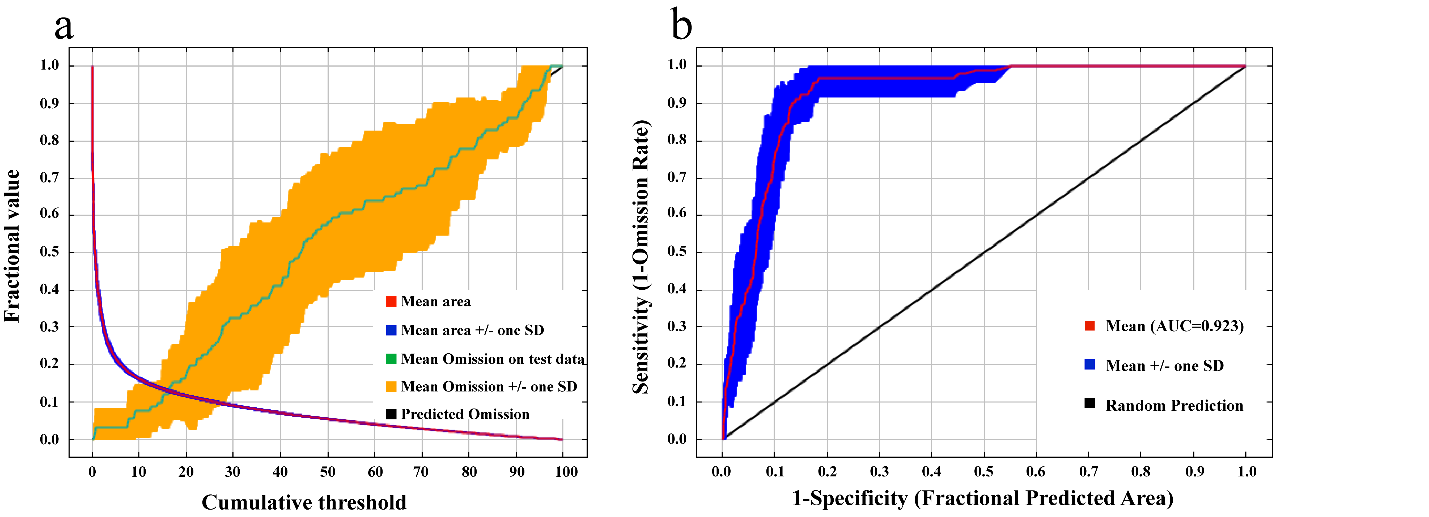


**Figure S5** Assessment of model prediction accuracy, (a) Average omission rate and predicted area as a function of the cumulative threshold, (b) Receiver operating characteristic (ROC) curve and AUC values under the current climate condition (10 replicated runs). The red curve indicates training data, the blue curve indicates test data, and the black line indicates random prediction, AUC, area under the curve.


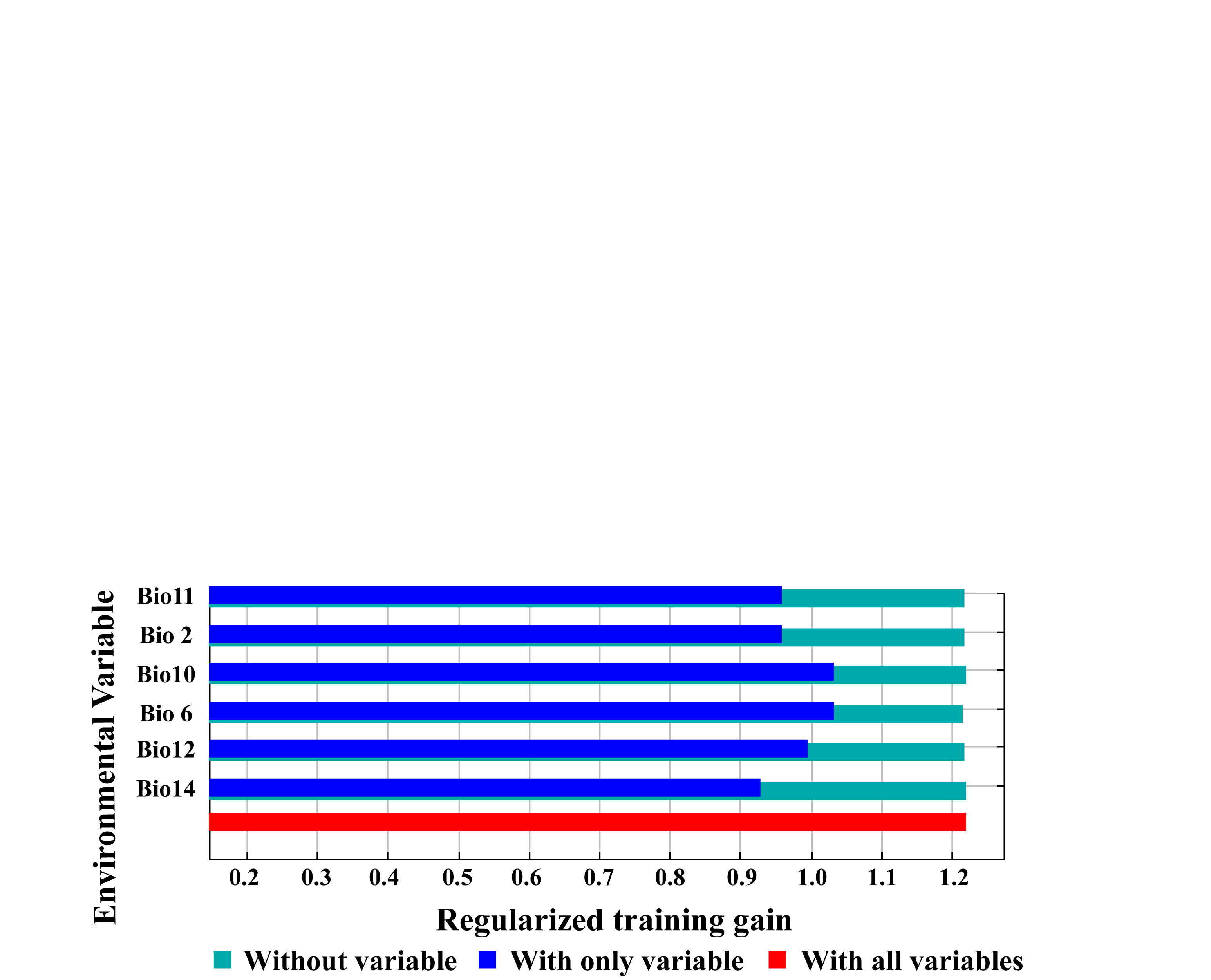


**Figure S6** Jackknife results of the regularized training gain in the maximum entropy models
